# Human parechovirus meningitis in children: state of the art

**DOI:** 10.1186/s13052-023-01550-4

**Published:** 2023-10-26

**Authors:** Elena Bozzola, Sarah Barni, Chiara Barone, Carlo Federico Perno, Andrea Maggioni, Alberto Villani

**Affiliations:** 1 Pediatric Unit, Bambino Gesù Children’s Hospital, IRCCS, Rome, Italy; 2 Multimodal Research Area, Microbiology and Diagnostics of Immunology Unit, Bambino Gesù Children Hospital IRCCS, Rome, Italy; 3 Nicklaus Children’s Hospital, Miami, FL USA

**Keywords:** Human parechovirus, Children, Meningitis, Central nervous system Infection

## Abstract

Human Parechovirus is a common cause of infection occurring especially during the first years of life. It may present with a broad spectrum of manifestations, ranging from a pauci-symptomatic infection to a sepsis-like or central nervous system disease. Aim of this study is to explore the knowledge on Parechovirus meningitis. According to the purpose of the study, a systematic review of the literature focusing on reports on central nervous system. Parechovirus infection of children was performed following PRISMA criteria. Out of the search, 304 papers were identified and 81 records were included in the revision dealing with epidemiology, clinical manifestations, laboratory findings, imaging, therapy and outcome. Parechovirus meningitis incidence may vary all over the world and outbreaks may occur. Fever is the most common symptom, followed by other non-specific signs and symptoms including irritability, poor feeding, skin rash or seizures. Although several reports describe favourable short-term neurodevelopmental outcomes at discharge after Parechovirus central nervous system infection, a specific follow up and the awareness on the risk of sequelae should be underlined in relation to the reported negative outcome. Evidence seems to suggest a correlation between magnetic imaging resonance alteration and a poor outcome.

## Introduction

Human Parechovirus (HPeV) is a common cause of infections occurring especially during the first years of life. It may present with a broad spectrum of manifestations, ranging from a pauci-symptomatic infection to a sepsis-like or central nervous system (CNS) disease, with possible neurological involvement, particularly among the youngest, that may even require intensive care unit assistance. The cytopathic effect, the rapid viral replication in neuronal cells, in combination with the likely lack of maternal protective antibodies and the immaturity of the immune system in toddlers may explain the potential danger related to HPeV infections in the youngest and the risk of sequelae [1].

Aim of the study is to review the current literature on HPeV meningitis in order to highlight the actual knowledge on epidemiology, clinical presentation, laboratory findings and imaging as well as therapeutic indication and need to follow up.

## Materials and methods

This systematic review followed the Preferred Reporting Items for Systematic Review and Meta Analysis (PRISMA) guidelines [2]. The literature research was performed through four different electronic databases: PubMed, Embase, Scopus and Web of Science, on 13th February 2023. For the aim of the study, the keywords used were “Parechovirus Meningitis”, and filters were added to limit the research to a paediatric population (< 18 years old), to reports written in English, and to limit the time spam to the last 5 years (2018–2023). Each research performed on each database was downloaded and then uploaded to the web tool “Rayyan web application” [3], a website used to analyse and appoint articles, specific for writing reviews.

### Eligibility criteria

To be included in the review, reports should satisfy the following inclusion criteria:

^th^ February 2023.

The exclusion criteria are:

− Issues not pertinent to the field of investigation;

− Reports including adults, without age distinction;

− Reports without data.

### Selection process

The selection process was conducted following the PRISMA guidelines, and it was assisted using the web application “Rayyan” [3].

First, the duplicates, produced by the research on four databases, were identified by the web application, Rayyan. Then, two authors checked the accuracy of the duplicates detected and excluded the unnecessary copies.

To limit errors and bias, two authors independently screened titles and abstracts produced by the research and defined those articles clearly irrelevant to the review. Afterward, full texts were retrieved and assessed for eligibility by the two screening authors. If full text articles couldn’t be found, an attempt of contacting authors was performed, to obtain the full text.

Finally, following PRISMA guidelines, references not included in the original search but relevant to the review were examined. Disagreements regarding inclusion/exclusion were settled through discussion between the researchers and a third author.

### Data Collection process and data items

Relevant articles were selected on the web application Rayyan and grouped together based on the different issues they dealt with.

Afterwards, data was compiled in a Microsoft Excel spreadsheet to evaluate the main topics reported in the last years about HPeV meningitis. The information extracted from the full-text reports included epidemiological, clinical, laboratories, radiological, therapeutic data, and outcome results.

### Data synthesis

Using the information gathered from the included studies, an updated review was achieved. The characteristics of the included studies were reported using descriptive statistics. No meta-analysis could be made with statistical work because of the variability of the studies. These results were then elaborated on in the discussion.

## Results

**Fig. 1:**
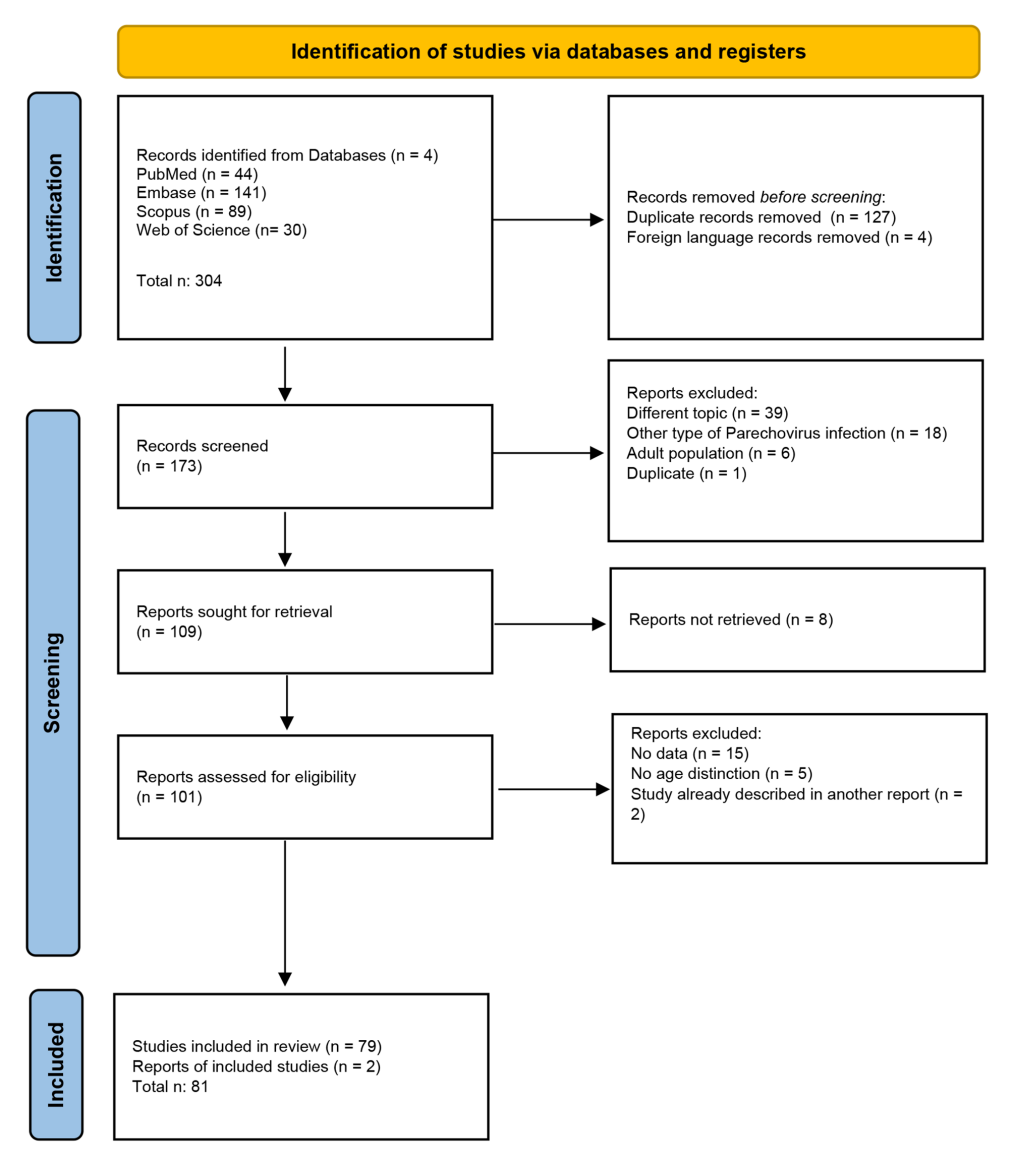
Flow chart of the selection process

The search of the selected electronic databases produced 304 studies. Diagram 1 presents the flow chart of the selection process, adapted from the PRISMA guidelines [4] (Fig. 1).

Out of them, 127 were the duplicates and 4 were not written in English. Then, according to PRISMA guidelines, all abstracts were analysed, and 63 records were discharged because they dealt with different topics, or with other types of HPeV infection, or with an adult population. Also, another duplicate article was found, not previously identified by the Rayyan web application.

Afterwards, 109 records were eligible to be analysed by reading their full-length text; however, 8 articles could not be retrieved. Therefore, 101 full-length reports were assessed for eligibility, and 21 were excluded because they did not display any data (n. 15), or no age subgroups could be found in a study population including adults and children (n. 5). In two cases the study reported had already been described in other articles. Finally, two relevant reports cited in other studies were added to this research.

In conclusion, 81 records were included in the revision.

Table 1 below shows the main issues found in this scoping review. Epidemiology was discussed in 39 reports, clinical manifestations in 47 reports, laboratory findings in 36 reports, imaging in 23 reports, therapy in 11 reports and outcome in 24 reports.

Table 2 displays all the reports included and their major findings.

**Table 1:** Main issues found in this scoping review

Issue	*n*	%
Epidemiology	39	48
Clinical Manifestation	47	58
Laboratory findings	36	44
Imaging	23	28
Therapy	11	14
Outcome	24	30

## Discussion

### Epidemiology

Of 81 reports analysed, 39 dealt with the epidemiology of HPeV meningeal infection [5–43].

#### Incidence

The reported percentage of HPeV positivity on Cerebrospinal Fluid (CSF) in children with meningeal involvement varied in American patients from 0.4 to 8.9%, with epidemic waves being occasionally reported [5–10]. HPeV type 3 was the most frequently detected single viral type [9].

As for Europe, many European Countries have been involved in epidemiological studies and highlighted the HPeV meningitis outbreak as well [11, 12]. The reported incidence in European patients varied as well from 0,04 to 10% [13–26].

Incidence of HPeV meningitis in the Asian continent has been studied mainly in Japan and Korea [27–34]. A multicentre study, conducted in Japan identified 240 infants with HPeV type 3 infection, of which 14.2% diagnosed with acute CNS infection [28]. Among 216 patients aged less than 4 months and hospitalised for fever, 110 were found to have a viral infection on serum or CSF, caused by HPeV in 60 cases [30]. In Korea the reported incidence varied from 8,6 to 37% [31–34].

As well as for other Continents, in Oceania incidence was varying from 5,4% to 25,8%, depending on the case series and period time considered [38–40].

**Fig. 2:**
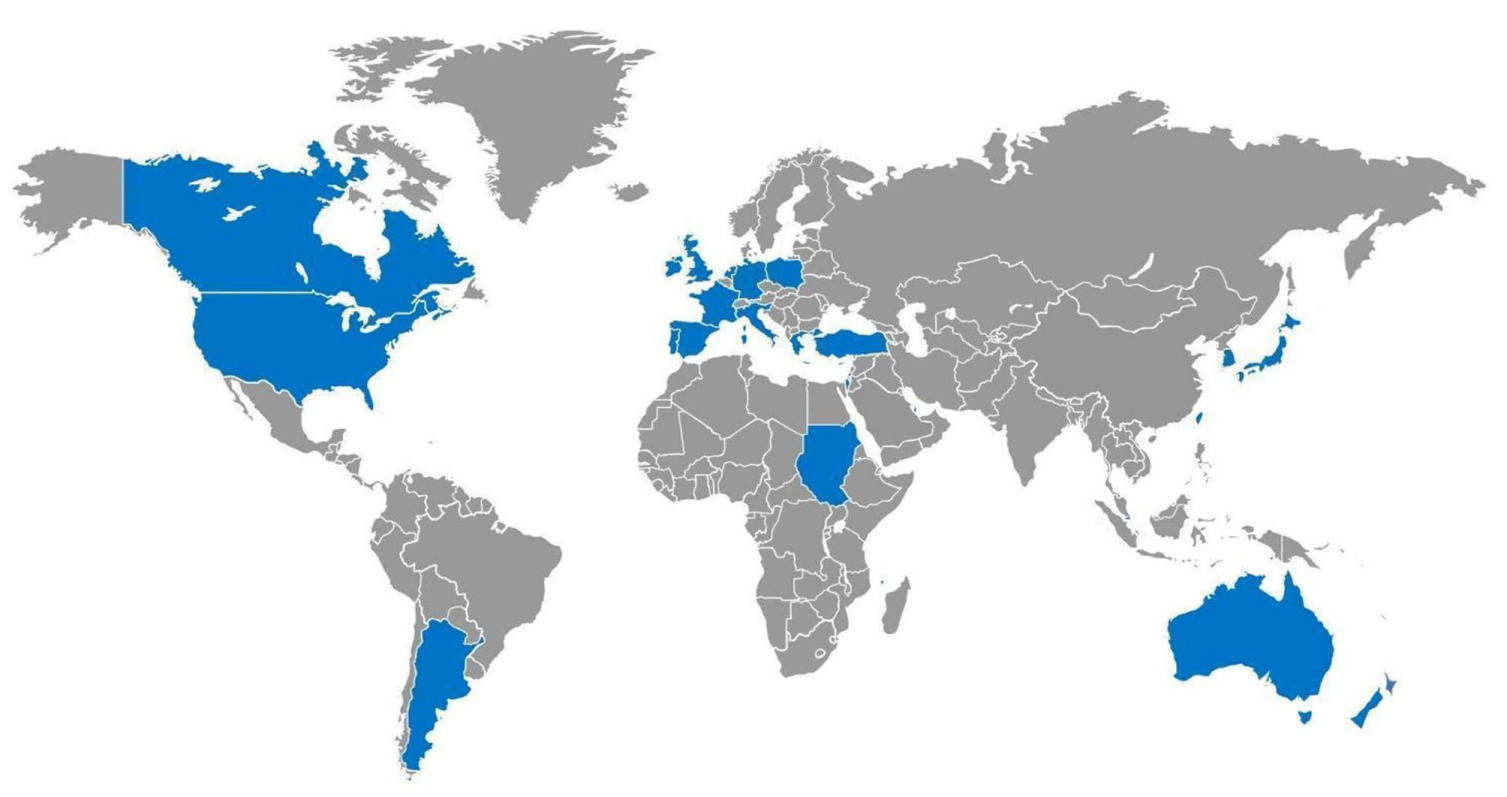
Countries involved in the studies are shown in blue on the World map. They were Argentina, Australia, Canada, Comoros, France, Germany, Greece, Ireland, Israel, Italy, Japan, Netherlands, New Zealand, Poland, Portugal, Qatar, Singapore, South Korea, Spain, Sudan, Taiwan, Turkey, United Kingdom., USA.

Incidence of HPeV meningitis in the African continent seemed to be low. In Sudan, between December and August 2010 no patient was found positive for HPeV on CSF, out of 503 children aged 0 to 15 years presenting with fever, seizures, and a suspicion of neuroinfection [42]. Nine years later in the Comoros archipelago, HPeV RT-PCR were performed on 122 CSFs, of which 77 were collected from children, and only a 30-days-aged infant presented with a CSF HPeV infection (0,8%) [43]. The Countries involved in the studies are represented on the Map in Fig. 2.

#### Seasonality

Some reports described the seasonality of the HPeV infection, with most of the cases presenting in the warmer months of the year [8–12, 18, 19, 27, 34, 43]. Whereas other studies didn’t find evidence of seasonality connected to HPeV meningitis [13–26].

#### HPeV genotypes

Regarding the molecular epidemiology of HPeV infection, HPeV type 3 was the predominant genotype, as reported by most studies analysed by this review [8, 9, 11, 17, 19, 21, 26, 27, 40]. Chamings A et al. described two cases of HPeV meningitis caused by the recombinant HPeV type 5 [41].

### Clinical manifestations

Of 81 reports analysed, 47 dealt with the clinical presentation of HPeV meningeal infection [1, 8–12, 14, 17, 21, 23, 25–28, 32, 33, 41, 44–73].

Almost all reports showed that the youngest (under 3 months of age and in particular neonates) were the most affected patients. [9–12, 14, 21, 23, 26–28, 32, 33, 59–62, 64–68, 70]. Of note, an exceptional adolescent onset was reported in an immunosuppressed 17-year-old girl [58].

Most studies reported a higher incidence in males (56.4–91%) than in females [9–11, 17, 21, 27, 59, 60, 62, 65, 66]. Only two Korean studies reported a female prevalence (57.1-80%) [32, 33].

Clinical presentation was nonspecific, mainly in neonates, so that the diagnosis may be challenging. At onset, patients appeared with sepsis-like symptoms and poor general conditions. Fever was the most frequent presenting sign [1, 41, 44–47, 49, 50, 52–58]. In fact, fever occurrence ranged between 80% and 100% [8, 10–12, 14, 17, 21, 23, 25–28, 32, 33, 60–64, 69, 70]. However, in an American retrospective study, 2% of patients reported hypothermia (TC < 35 °C) instead of hyperthermia [68].

Other frequent symptoms included irritability, from 40 to 100%, poor feeding, from 42 to 81.8%, and tachycardia ranging from 63.6 to 77% [12, 14, 26, 41, 44–46, 51–54, 58, 60, 62–64, 67–70]. In a few cases lethargy was the presenting symptom, with a prevalence between 14% and 51% [1, 12, 14, 26, 60, 69].

Many clinical cases of HPeV CNS infection included a cutaneous sign, as macular or maculo-papular rash, involving mainly truncus and extremities [14, 23, 41, 45, 47, 54–58, 62]. The occurrence of a rash as a presenting sign ranged from 20 to 60% [1, 8, 11, 12, 17, 21, 52, 53, 60, 64, 68, 69].

Moreover, 15 reports evidenced the presence of seizures [1, 10, 11, 21, 26, 28, 45, 50, 58, 62, 65–68, 70]. Their occurrence may vary from 3.1 to 65.2% [10, 11, 21, 26, 28, 62, 65–68, 70].

Gastrointestinal symptoms were described as concomitant to meningitis, usually as diarrhoea, with a variable occurrence, ranging from 15 to 30% [12, 23, 52].

Other symptoms involving the respiratory system have been described, including coryza, cough, breathing difficulties, apnea and tachypnea. Their reported occurrence varied from 37.5 to 18.2% [11, 64, 68, 70].

#### Congenital HPeV Infection

Three cases of congenital/in utero transmission had been described in recent literature, leading to neonatal meningitis at birth, requiring intensive care unit support. The onset presentation symptoms were hypotonia, respiratory distress with desaturation, bradycardia, fever and abnormal movements [71–73].

### Laboratory findings

Out of the 81 articles included in this review, 36 focused on the laboratory findings [1, 8, 12, 14, 17, 18, 21, 23, 26, 27, 31, 33, 36, 38, 41, 44, 45, 48, 51–53, 55–57, 61–63, 67, 69, 70, 72–76].

#### Blood analysis

In children with HPeV meningitis, peripheral leukocyte count, haemoglobin and platelets were within normal value range [51, 52]. Leukopenia was found especially in neonates and infants aged < 3 months [1, 55, 63, 67]. Low values of haemoglobin were occasionally described [12, 56].

General chemistry was unremarkable [41], although some authors described an increase of the transaminase, lactate dehydrogenase (LDH) values and hyponatremia [27, 51, 67, 70].

Inflammation markers were generally in the range of normality or mildly elevated [1, 12, 18, 31, 41, 52, 53, 55–57, 63, 69, 70]. Of note, compared with Enterovirus (EV) positive infants, infants with HPeV meningitis had lower values of blood white blood cells [27, 33] and infection indices [17].

Blood cultures were reported as negative [1, 41, 48, 62].

#### CSF analysis

CSF samples of children with HPeV meningitis were clear and pleocytosis characteristically mild or absent [1, 8, 12, 14, 21, 26, 27, 36, 38, 41, 45, 51, 53, 55, 57, 62, 67, 69, 70, 72, 74].

Despite that, some case-reports described pleocytosis in children, predominantly neonates, with HPeV meningitis [44, 48, 57, 61, 73]. Compared with EV meningitis, HPeV meningitis determined a lower rate of CSF pleocytosis [14, 17, 27, 32, 33, 61]. In case of pleocytosis, white cell count was lower in the HPeV meningitis than in the EV group, and other viral meningitis, such as Human Herpes 6 and Herpes Simplex Virus-2 meningitis [23, 32].

As for the remaining CSF biochemistry, in HPeV meningitis proteins were normal or slightly elevated [1, 8, 14, 18, 21, 27, 45, 55, 56, 61, 62, 70, 72]. Moreover, glucose values were generally normal [1, 8, 21, 27, 41, 51, 55, 61, 62, 70, 72]. Of note, low glucose concentration has been described just in few cases, mainly in neonates [57, 63].

CSF cultures were negative [1, 41, 45, 48, 51–53, 62].

#### Cytokine Profile on serum and CSF

Measurement of cytokines levels revealed high levels of interleukins (IL): IL-6, IL-17 and TNF alpha on serum and of IL-2, IL-4, IL-7 and IL-13 in CSF [31, 75].

Compared to EV, a higher serum level of proinflammatory cytokine/chemokine was present in HPeV, likely related to the more severe clinical manifestations in the former [76].

### Imaging

HPeV meningitis was diagnosed by clinical manifestations and laboratory findings. Instrumental diagnostic exams were used as complementary and to investigate HPeV’s clinical complications and outcomes. Twenty-three publications analysed instrumental exams in patients affected by HPeV meningitis [1, 10, 12, 25, 26, 28, 45, 48–50, 56–58, 62, 65–68, 71–73, 77, 78].

Head ultrasound and cerebral magnetic resonance imaging (MRI) were the most used diagnostic exams; few cases reported head Computerised Tomography (CT) scan finding.

#### Head ultrasound

Head ultrasound was used in reports as it is a fast, non-invasive and economic diagnostic exam. Nevertheless, it generally resulted in normal finding [1, 12, 25, 45, 48, 50, 56, 62, 77, 78].

#### Magnetic resonance imaging

When performed in patients with HPeV neuro-infections, MRI resulted in white matter anomalies. Common findings included restricted diffusion in deep white matter and periventricular white matter involving mainly the frontal zones [48,50,65, 6872]. Involvement of parietal and temporal lobes, corpus callosum and thalamus have been described as well [58, 71, 73, 77, 78]. Hyperintensity in the T2/FLAIR was also a possible presentation of HPeV neuro-infection [28]. These findings were typically bilateral, either asymmetrical or symmetrical [50, 58, 65]. Other findings, such as unilateral lesions, low signal intensity on T2 and hyperintensity on T1 or just a leptomeningeal enhancement were also described [28, 50].

Some authors reported that MRI abnormalities were usually detected in a minority of children with HPeV CSF infections [1, 10, 12, 26, 57, 65]. Conversely, other authors demonstrated that the majority of patients in the study populations had positive MRIs findings [28, 66].

There’s evidence that most of the patients with white matter alterations on MRI developed more severe diseases, with seizures or necessity of ventilations and vasoactive infusion [67].

When performed weeks or months after the acute infection, MRI scan might be normal, with no evidence of white matter lesions [49, 58]. Persistence of the lesions had been proved in a minority of patients [28, 65].

White matter MRI anomaly had been also used as a prognostic sign of neurodevelopmental concerns. By the way, some authors found neurodevelopmental impairment at clinical follow-up in children with initial MRI alterations [28, 65, 66].

Bucci S. et al. showed that children with MRI abnormalities in the HPeV acute infection scored lower, but still in the range of normality, on cognitive Bayley III (Bayley Scales of Infant and Toddler Development, Third Edition) subscale at Neurodevelopmental assessment at 1 year of age compared with children with normal MRI [25]. Whereas Abe Y. et al. reported that the totality of the patients (6/6) with MRI negative findings had a neurological good prognosis [28].

#### Other investigations

Electroencephalography (EEG) demonstrated seizure activity or encephalopathy signs [67, 68, 72,73,7868].

### Therapy

Out of the included reports, eleven focused on the therapeutic approach [10, 21, 45, 50, 56, 57, 62, 68, 69, 73, 79]. Authors agree on supportive therapy, including paracetamol and fluids. Antibiotics as well as antivirals were prescribed at onset and then discontinued as soon as the diagnosis of HPeV infection was confirmed [21, 56, 57, 62, 69, 73].

In case of seizures, antiepileptic therapy was considered [10, 45, 68, 73].

In case of critical conditions or unresponsiveness to therapy, intravenous immunoglobulin and/or methylprednisolone have been prescribed [10, 21, 50, 69]. Finally, posaconazole was used against HPeV type 3, acting as an early-stage inhibitor of viral replication: it binds the capsid interfering with virus-cell attachment and entry [79].

### Outcome

We identify 24 publications dealing with clinical outcome of HPeV meningitis, in term of hospital stay and long-term outcome [1, 12, 21, 25, 26, 28, 44, 48, 49, 56, 57, 59, 60, 62, 65, 66, 70, 73, 74, 80–84].

#### Hospital Stay

Hospitalisation has been analysed in 9 reports [1, 12, 21, 48, 49, 56, 57, 62, 70].

Hospitalisation ranged from 2 days to 5 weeks. The high variability was depending on various factors, including the clinical course and the need of intensive care in case of respiratory distress, apnoea, seizures, and hemodynamic instability [1, 12, 21, 48, 49, 56, 57, 62, 70].

All but one otherwise healthy 11-days old neonate affected by HPeV type 3 meningoencephalitis survived. The neonate’s autopsy showed bilateral multicystic cavitation of the fronto-parietal white matter as well of temporal and occipital asymmetric cavitation [78].

#### Long-term outcome

The long-term outcome was analysed in 21 reports.

Even if HPeV is one of the main identified etiological agents of viral meningitis in infants, poor attention has been reserved to it from the Scientific Community in the past, mainly due to the high survival range. Examining scientific reports from all over the world are increasing awareness of the risk connected to a severe parechovirus infection. In fact, out of the revised literature, cerebral palsy, vision and neuropsychomotor development impairment were reported in a high percentage of case series [65, 80, 83]. The appropriate duration of post hospitalisation follow-up is still being debated. Evidence supported clinical follow-up until at least the second year of life, with a recommended longer-term follow-up in case of further potential risk factors, such as prematurity, early onset of infection (neonatal period), MRI abnormalities, severe clinical course (seizures, apnoea) with necessity of paediatric Intensive Care Unit [1, 25, 28, 48, 65,66]. Evidence suggested an ameliorating of clinical sequelae with a normal development in most cases by the age of three [65]. Anyway, as neurodevelopmental impairment is often difficult to detach at an early stage, prior to school-age, families should be aware of potential neurological, behavioural, and learning impairments in childhood in order to eventually seek assessment.

## Conclusion

HPeV infection is very common in paediatric age and may have a severe course mainly among neonates and toddlers less than 3 months of age when it manifests as meningitis. Symptoms may be non-specific, including fever, irritability, poor feeding, skin rash, or seizures. Although several authors described favourable outcome with high probability of survival, reported neurodevelopmental outcomes at discharge suggest a specific follow up and the family awareness on the risk of sequelae.

Evidence supported clinical follow-up until at least the second year of life, with a recommended longer-term follow-up in case of further potential risk factors, such as prematurity, early onset of infection (neonatal period), MRI abnormalities, severe clinical course (seizures, apnoea) with necessity of paediatric Intensive Care Unit. Of note, we emphasise the need for surveillance to define the disease burden, evaluate strategies and interventions to prevent and manage cases, and to respond to the potential first early signals of sequelae developing. Finally, defying and following the global epidemiology of HPeV infection may be useful for considering the opportunity of vaccine development mainly for those with risk factors for a severe course.

**Table 2:** Reports included and the major finding

Domains	Reference	Type of Publication	Country (Continent)	Highlighted
Clinical Presentation Laboratory Imaging Outcome	Ancora G, 2020 [1]	Case Report	Italy (Europe)	Six neonates presented with fever and poor feeding, rash in 3 cases, seizures in one. Blood analyses revealed lymphopenia, normal or mildly elevated CRP, no pleocytosis. Head ultrasound and brain MRI were abnormal only in one case. The median hospitalisation was 11 days (range 7–26). At 2 years follow up just one developed epileptic encephalopathy, vision and psychomotor development impairment
Epidemiology	Naccache SN, 2018 [5]	Retrospective study	USA (America)	In the period study, out of 251 patients, 0.4% of CSF was positive for HPeV
Epidemiology	Abedi GR, 2019 [6]	Retrospective multicentre study	USA (America)	In the period study, 3.4% of children under the age of 1 year had CSF positive for HPeV
Epidemiology	Lee BR, 2020 [7]	Retrospective cohort study	USA (America)	In the period study, out of 1926 specimens of CSF in children less than 6 months of age, 8.9% were positive for HPeV
Epidemiology Clinical Presentation Laboratory	Tomatis Souverbielle C, 2021 [8]	Retrospective study	USA (America)	In the period study, out of 1475 infants younger than 60 days, 130 were HPeV positive. Infections mainly occurred in summer. Out of them, 96% had fever, 75% fussiness, and 29% a rash. In CSF, protein and glucose levels were within the normal ranges; 77% had no CSF pleocytosis. HPeV type 3 was the only one detected in CSF
Epidemiology Clinical Presentation	Sasidharan A, 2021 [9]	Single-site study	USA (America)	In the period study, 7% (271/4,016) CSF were positive for HPeV. 95% was HPeV type 3. Most infections occurred during summer. Most patients were males (169/271; 62%) with a median age of 27.2 days, only six (2%) were 4-6-month-old
Epidemiology Clinical Presentation Imaging Therapy	Hardeep Singh S, 2023 [10]	Retrospective study	USA (America)	In the period study, out of 14 patients with HPeV on CSF, 43% were infected in summer-fall and 57% in spring. Median age was 21 days, 57% were male. Symptoms were fever and fussiness (100%), poor oral intake (50%), seizures (7%). One out of 4 patients had abnormal MRI findings. Anti-epileptics medicines, corticosteroids and intravenous immunoglobulin were used in 2 patients with seizures. All the others received supportive care.
Epidemiology	Black S, 2019 [11]	Original research	UK (Europe)	In the period study, 32 CSF specimens were HPeV positive, all genotype 3, with an outbreak in summer 2016. Patients had 25.5–61 days, 59.4% were male, 100% had fever, 68.8% poor feeding, 25% rash, 3.1% seizures, 37.5% respiratory symptoms (coryza, cough, wheeze)
Epidemiology Clinical Presentation Laoratory Imaging Outcome	Antolin LF, 2018 [12]	Original research	UK (Europe)	HPeV infection’s highest peak was in summer. 43% were neonates; 42% were less than 90 days. Symptoms were: fever (92%) irritability (63%), reduced feeding (49%), rash (25%), lethargy (23%), diarrhoea (15%). 7% of infants had anaemia, 14% lymphopenia, 4% neutropenia and 4% thrombocytopenia, 75% low CRP level. On CSF, WCc was higher than 20 cell/mcL in 8% of neonates and higher than 5 cell/mcL in 7% of infants. Head ultrasound was normal in 10 infants; MRI scans were normal in 5/7, compatible with encephalitis in 1/7 and with brain oedema in 1/7. Median hospitalisation was 3 days (range 2–11) After discharge, 5 infants had ongoing seizures and 1 neurological impairment
Epidemiology	Cosgun Y, 2020 [13]	Retrospective study	Turkey (Europe)	In the period study, out of 5255 CSF samples, two were HPeV positive
Epidemiology Clinical Presentation Laboratory	Chakrabarti P, 2018 [14]	Retrospective study	UK (Europe)	In the period study, out of 140 CSF samples, 10% tested positive for HPeV. Out of them, 85% were less than 2 months of age, 100% had fever, 42% irritability, 14% lethargy, 42% a maculo-papular rash. No CSF sample showed pleocytosis and just 21.4% had increased CSF protein levels. HPeV meningitis had lower CSF WCc than EV meningitis
Epidemiology	Sirin MC, 2018 [15]	Retrospective cross-sectional study	Turkey (Europe)	In the period study, out of 59 CSF specimens, 1.7% resulted HPeV positive
Epidemiology	Bal A, 2022 [16]	Multicentre cross-sectional retrospective study	Turkey (Europe)	In the period study, out of 179 CSF samples, two were HPeV positive
Epidemiology Clinical Presentation Laboratory	De Jong EP, 2018 [17]	Prospective observational cohort study study	The Netherlands (Europe)	In the period study, out of 353 samples of infants aged less than 90 days, 39 were HPeV positive; type 3 was the only genotype found. Out of them, 46% were neonates, 62% were male. 100% had fever and 20% a rash. Compared to EV, HPeV meningitis have lower infection indices and lower rate of CSF pleocytosis
Epidemiology Laboratory	Eichinger A, 2019 [18]	Retrospective study	Germany (Europe)	In the period study, out of 187 CSF samples, two were HPeV positive, occurring in May and September. CSF WCc had a mean value of 10 +/- 4 cell/mcL. CSF Protein level was normal
Epidemiology	Elling R, 2019 [19]	Original research	Germany (Europe)	In the period study, out of 11 CSF of 25 HPeV infected patients, 8 tested positive for HPeV type 3. Most of the infections occurred in summer
Epidemiology	Posnakoglou L, 2020 [20]	Prospective cohort study	Greece (Europe)	In the period study, out of 142 CSF samples, four were HPeV positive (2.8%)
Epidemiology Clinical Presentation Laboratory Therapy Outcome	Posnakoglou L, 2021 [21]	Prospective cohort study	Greece (Europe)	In the period study, out of 330 CSF specimens, 6 were HPeV positive (1.8%); type 3 was the only genotype. Patients were all under 3 months of age, 83% males. 100% presented with fever, 50% rash, and 16% seizures. No pleocytosis and normal levels of protein and glucose were found in CSF. All received antibiotics and one neonate also had intravenous immunoglobulin. Hospitalisation was 7.5 days long (IQR: 5.3–10.3)
Epidemiology	Vincent JJ, 2020 [22]	Original research	France (Europe)	In the period study, out of 309 CSF samples, 12 were HPeV positive
Epidemiology Clinical Presentation Laboratory	Marchand S, 2021 [23]	Retrospective study	France (Europe)	In the period study, out of 1373 CSF specimens, 34 tested positive for HPeV. No seasonality was observed. 97% were under 3 months of age, 100% presented with fever, 13% had an erythematous and maculopapular rash, 23% had diarrhoea. Pleocytosis was observed in 11% of cases, lower than in case of EV meningitis
Epidemiology	Schnuriger A, 2022 [24]	Original research	France (Europe)	In the period study, out of 1,744 CSF samples, 11 cases of HPeV infection were identified, and they all occurred in neonates
Epidemiology Clinical Presentation Imaging Outcome	Bucci S, 2022 [25]	Original research	Italy (Europe)	In the period study, 3 out of 15 CSF samples of newborns with viral meningitis were HPeV positive. All presented with fever. Head ultrasound was normal in all patients, MRI in 1. At 1 year follow up, 29 out of 30 patients had no sequela. Just one child had a mild delay in fine and gross motor skills and in receptive language
Epidemiology Clinical Presentation Laboratory Imaging Outcome	Stephens C, 2021 [26]	Retrospective study	Ireland (Europe)	In the period study, there were 23 cases of CSF HPeV positive, all younger 2 months of age; 90% were serotype 3. No seasonality was found. Out of them, 90% was febrile, 40% irritable, 20% lethargic, 10% had seizures. None had CSF pleocytosis. Just 25% showed frontoparietal white matter changes at MRI. At 3 years follow up, 60% of patients had a normal development
Epidemiology Clinical Presentation Laboratory	Sano K, 2018 [27]	Prospective study	Japan (Asia)	In the period study, over 56 febrile infants, 2 tested positive for HPeV on CSF. HPeV infections had a summer peak. Fever was the symptom of presentation. WBC count, platelets values and CSF pleocytosis rate was lower than in EV
Epidemiology Clinical Presentation Imaging Outcome	Abe Y, 2021 [28]	Retrospective study	Japan (Asia)	In the period study, 34 out of 240 infants with HPeV infection had acute CNS involvement. 87% were younger than 2 months of age, 87% presented with fever and 65.2% with seizures. 57.1% presented with lesions on 15 months follow-up MRI. Half patients with abnormalities at MRI follow-up had neurological sequelae. Patients with normal MRI follow-up had no neurological sequelae
Epidemiology	Izumita R, 2019 [29]	Prospective study	Japan (Asia)	In the period study, over 54 febrile neonates and young infants, HPeV was detected in serum and/or CSF of 14 patients
Epidemiology	Suziki Y, 2021 [30]	Prospective study	Japan (Asia)	In the period study, 60/216 infants were found to have an HPeV infection on serum or CSF. HPeV type 3 was detected in 93% of cases, HPeV type 4 in 5% and 2% of untyped virus
Epidemiology Laboratory	Park SE, 2019 [31]	Prospective cohort study	Korea (Asia)	In the period study, out of 90 children less than 1 year) of age with fever and sepsis-like signs, 10 had HPeV meningitis. In the CSF there was a significantly higher level of IL-2, IL-4, IL-7 and IL-13, than in controls
Epidemiology Clinical Presentation Laboratory	Park SE, 2021 [32]	Retrospective cohort study	Korea (Asia)	In the period study, HPeV was identified in 10 CSF samples out of 110. 70% aged younger than 2 months, 20% were male. All presented with fever, 20% with seizures. Compared to other viral meningitis, CSF samples had the lowest value of white cell count
Epidemiology Clinical Presentation Laboratory	Rhie S, 2020 [33]	Retrospective study	Korea (Asia)	In the study period, 14 out of 161 CSF samples were HPeV-positive. All patients were younger than 3 months of age, 42.9% were male. All had fever. Compared to EV meningitis, patients had lower WCB count and lower rate of CSF pleocytosis
Epidemiology	Nam EJ, 2021 [34]	Retrospective study	Korea (Asia)	In the study period, over 2230 CSF samples, 10 were positive for HPeV. They all aged less than 3 months. HPeV was found only in summer
Epidemiology	Chang JT, 2018 [35]	Retrospective study	Taiwan (Asia)	In the study period, 1 out of 112 children aged less than 10 years and positive for HPeV was affected by HPeV meningitis
Epidemiology Laboratory	Nassrallah B, 2021 [36]	Retrospective study	Israel (Asia)	In the study period, 5 out of 80 febrile infants aged less than 3 months had CSF positive for HPeV. Pleocytosis was absent in all
Epidemiology	Mathew S, 2021 [37]	Retrospective study	Qatar (Asia)	In the study period, HPeV was isolated in 26 out 30 children aged less than 9 years
Epidemiology Laboratory	O’Brien MP, 2018 [38]	Retrospective and prospective cohort study	Australia (Oceania)	In the study period, 8 out 65 children aged 0–16 years with meningitis were HPeV positive. Five cases were aged less than 3 months. No pleocytosis was found
Epidemiology	Berkhout A, 2023 [39]	Retrospective study	Australia (Oceania)	In 2o19, 5/93 infants’ CSF samples tested HPeV positive; in 2021 no HPeV was detected in CSF samples
Epidemiology	Chamings A, 2019, [40]	Original Reserach	Australia (Oceania)	In the period study, 26 out of 33 infants affected by HPeV had meningitis; the only genotype was HPeV3
Epidemiology Clinical Presentation Laboratory	Chamings A, 2019 [41]	Retrospective study	Australia (Oceania)	6 cases of HPeV meningitis were reported in children aged 14–68 days, all caused by HPeV type 5. Symptoms were irritability, fever, tachycardia, erythematous rash, poor feeding, irritability, dyspnoea. Blood analysis on 2 cases showed respectively WBCc of 5700 and 6500/mmc, CRP not elevated in the first case and mildly elevated in the second. CSF protein values were respectively normal and mildly elevated
Epidemiology	Abdelrahim NA, 2022 [42]	Cross-sectional study	Sudan (Africa)	Between December and August 2010, 0/503, febrile children tested positive for HPeV in CSF
Epidemiology	Fourgeaud J, 2022 [43]	Retrospective study	Comoros (Africa)	Between March and June 2019 only 1/122 CSF tested positive for HPeV, collected from a 30-days-aged infant in early June
Clinical Presentation Laboratory Outcome	Garrido R, 2022 [44]	Case Report	Portugal (Europe)	An 11-day-old infant with fever, irritability, and poor feeding, CSF pleocytosis and increased protein level recovered well. At 18 months follow up, she was in health
Clinical Presentation Laboratory Imaging Therapy	Fox B, 2022 [45]	Case Report	Argentina (America)	In the reported three cases, symptoms were fever, decreased oral intake, irritability, rash, seizures, bradycardia, desaturation, abdominal tenderness. CSF was clear and colourless, pleocytosis absent. Mild proteinorraquia was reported in one case. In just one case head ultrasound showed subcortical white matter hypoechogenicity of both cerebral hemispheres, corpus callosum, and both temporal lobes. Intravenous antibiotics and antiepileptic medicines were administered.
Clinical Presentation	Alhazmi A, 2020 [46]	Case Report	France (Europe)	One-month-old baby girl with high fever, irritability, vomiting, and diarrhoea for 36 h
Clinical Presentation	Chowdhury SR, 2020 [47]	Case Report	Singapore (Asia)	3-month-old infant with fever, tachycardia, acral swelling and maculo-papular rash
Clinical Presentation Laboratory Imaging Outcome	Berk MC, 2018 [48]	Case Report	The Netherlands (Europe)	A male premature neonate born at 32 weeks and 6 days gestational age presented with sepsis-like symptoms, elevated serum CRP, mild pleocytosis, low level of proteins and normal values of glucose in CSF. Head ultrasounds was normal, MRI showed bifrontal periventricular abnormalities of the white matter. He was discharged at 37 weeks and 6 days of life. At 5 years follow up the patient presented with cerebral palsy, vision, and psychomotor development impairment
Clinical Presentation Imaging Outcome	Piralla A, 2019 [49]	Case Report	Italy (Europe)	A 15-day-old boy with high fever, poor feeding, inconsolability, tachycardia, and mild axial hypotonia had a negative head ultrasound and a normal brain MRI at a 2 months follow up. Hospitalisation lasted 7 days
Clinical Presentation Imaging Therapy	Yehia R, 2023 [50]	Case Report	USA (America)	Two neonates presented with fever, fussiness, seizures, apnoea, and cyanosis. They had normal head ultrasound and head CT. Patient 1 had normal brain MRI findings. Patient 2 showed restricted diffusion throughout the bilateral white matter and leptomeningeal enhancement on MRI. Both patients received intravenous immunoglobulin and corticosteroids.
Clinical Presentation Laboratory	Kirkley MJ, 2019 [51]	Case Report	USA (America)	A 15-day-old infant with fever, fussiness, irritability, poor feeding, grunting respirations and distended abdomen had normal WBC count, in CSF normal WBC count, glucose and normal proteins
Clinical Presentation Laboratory	Kanagaratnam M, 2019 [52]	Case Report	UK (Europe)	7 neonates presented with fever and severe irritability, 60% with rash, and 30% with diarrhoea. Blood WBC count was normal, and CRP was low in 90% of cases. CSF tests showed normal WBC count, protein, and glucose levels
Clinical Presentation Laboratory	Urooj F, 2019 [53]	Case Report	UK (Europe)	4 patients aged 1–10 weeks, 50% female, had high fever, sepsis-like-symptoms, and irritability, 50% a maculo-papular rash. CRP serum values were normal or slightly elevated. CSF samples had a normal WBC count
Clinical Presentation	Lee D, 2018 [54]	Case Report	Singapore (Asia)	2-month-old girl with fever, irritability, and maculopapular exanthema. On day three, she developed nontender palmoplantar erythema
Clinical Presentation Laboratory	Masanori T, 2022 [55]	Case Report	Japan (Asia)	A 2-month-old girl with fever, poor general condition, severe abdominal distention, rash and acral foot reddening showed low values of WBC count and negative CRP. CSF examination showed normal results
Clinical Presentation Laboratory Imaging Therapy Outcome	Tokmak DN, 2021 [56]	Case Report	Turkey (Europe)	A 38-day-old infant with sepsis-like condition, fever and rash had normal WBC count on CSF and blood, low haemoglobin value, normal biochemical parameters, and CRP mildly elevated. Head ultrasound was normal. He received intravenous antibiotics and acyclovir. hospitalisation was 6 days long
Clinical Presentation Laboratory Imaging Therapy Outcome	Tokak S, 2021 [57]	Case Report	Turkey (Europe)	A 43-day-old girl with HPeV meningitis presenting with fever, sucking weakness and petechial rashes. She had slightly elevated serum CRP. CSF analysis showed a clear fluid, lack of pleocytosis and low levels of glucose. Antibiotic therapy was administered. Brain MRI was normal. Hospitalisation lasted 8 days
Clinical Presentation Imaging	Tierradentro-García LO, 2022 [58]	Case Report	USA (America)	15-day-old boy with confluent erythematous rash on trunk, poor feeding, irritability, fever, seizures of the right arm and rightward eye deviation; 11-day-old boy with fever, irritability, apnoea episodes, and right-sided clonic seizure; 17 years-old girl, in immunosuppressive therapy, with fever and left-side weakness, unsteady gait, headache. Head ultrasonography showed increased echogenicity of the periventricular WM. Brain MRI showed restriction of diffusion in WM matter, with a frontoparietal predominance
Clinical Presentation Outcome	van Hinsbergh T, 2022 [59]	Prospective multicentre study	The Netherlands (Europe)	The median age of onset is 29 days; 91% are males. In the 5-year-follow-up, patients had gross motor impairment
Clinical Presentation Outcome	van Hinsbergh TMT, 2019 [60]	Prospective multicentre study	The Netherlands (Europe)	9 patients with a median age of 31 days, 89% male had fever (100%), irritability (77.8%), poor feeding (55.6%), lethargy (22.2%), rash (33.3%) At 6 months follow up, 6 had a gross-motor function delay
Clinical Presentation Laboratory	Tan JHY, 2022 [61]	Retrospective study	Singapore (Asia)	71 children younger than one year, presented with fever, and 42% with poor feeding. Out of them, 2.8% presented with CSF pleocytosis, median CSF level of proteins of 0.43 g/L and median value of glucose 3.2 mmol/L
Clinical Presentation Laboratory Imaging Therapy Outcome	Linhares MI, 2021 [62]	Original research	Portugal (Europe)	7 neonates, of which six males, presented with fever and irritability, two with acral involvement rash, two with gastrointestinal symptoms, and one with seizures. In CSF no pleocytosis, protein and glucose normal values were found. Head ultrasound was normal. Empirical intravenous antibiotics were started in six infants, until negative culture results were obtained. Hospitalisation had a mean of 5.6 days (3–11 days). Outcome was favourable.
Clinical Presentation Laboratory	Kielar M, 2019 [63]	Retrospective study	Poland (Europe)	Neonatal HPeV CNS infections all presented with fever and irritability. Analysis revealed leukopenia, low inflammatory index. On CSF, low glucose values were found in 3 patients and increased cell count was found in 7 patients
Clinical Presentation	Samarasekara H, 2020 [64]	Original research	Australia (Oceania)	5 cases, two-six weeks of age. All with fever and sepsis-like symptoms; four were irritable and one presented desaturation and tachypnoea
Clinical Presentation Imaging Outcome	Silcock RA, 2022 [65]	Prospective cohort study	Australia (Oceania)	Median age was 27 days, 62.5% were male, 7.8% had seizures, 32.5% abnormal findings on brain MRI (bilateral white matter signal changes and periventricular restricted diffusion patterns). In the second year of life, 63% (29/46) children showed developmental delay, which ameliorated by the third year of life (30%). Communication was the most common domain of concern
Clinical Presentation Imaging Outcome	Joseph L, 2019 [66]	Retrospective study	Australia (Oceania)	Out of 77 patients, 87.5% were under 3 months, (37.2% neonates) and 56.4% male. 9.7% of patients had seizures. 15 out of 20 children had abnormal MRI findings (restricted diffusion in deep white matter); at follow-up 11 (14%) had neurodevelopmental delay
Clinical Presentation Laboratory Imaging	McKenna R, 2019 [67]	Retrospective multicentre study	Australia (Oceania)	Patients admitted to ICU were all younger than 3 months, 93% had irritability, 57% apnoea, and 40% seizures. Lymphopenia was found in most of the cases. CRP values were low or mildly elevated. 27% of patients had hyponatremia and 70% had increased transaminase values. Pleocytosis was absent, CSF protein level was normal and glucose values ranged from 2.3 to 6.7 mmol/L. 78% of patients with white matter alterations on MRI developed severe diseases
Clinical Presentation Imaging Therapy	Midgley CM, 2018 [68]	Retrospective study	USA (America)	35 neonates presented with 91% fever, 2% hypothermia, 77% tachycardia, 77% poor feeding, 74% irritability, 26% rash, 20% seizures and 9% breathing difficulties. Brain MRI was performed in 3 infants who presented seizures, showing white matter abnormalities, EEG severe encephalopathy and multifocal epileptiform discharges in 2 cases. Acyclovir and anticonvulsant medication were administered in patients with seizures.
Clinical Presentation Laboratory Therapy	Kadambari S, 2019 [69]	Prospective study	UK (Europe)	Out of 35 patients, 80% had fever, 66% poor feeding, 66% irritability, 51% lethargy, 29% rash. CSF WBC count was less than 20/mm^3^, serum CRP was low in 67% of cases.Empiric intravenous antibiotics were administered. 46% of patients received acyclovir. One patient received intravenous immunoglobulin.
Clinical Presentation Laboratory Outcome	Roh DE, 2020 [70]	Retrospective study	Korea (Asia)	11 patients, median age of 41 days, presented with fever (100%), poor feeding (81.8%), irritability (63.6%), tachycardia (63.6%), tachypnoea (36.4%), apnoea (18.2%), seizures (9.1%). Blood tests showed a mean WBCc value of 5622/mmc. CRP e procalcitonin were low. Hyponatremia and mild transaminasemia were reported. Pleocytosis was absent. Protein and glucose values in CSF were normal. Hospitalisation was of 7.1 ± 3.6 days
Clinical Presentation Imaging	Hilbig, A, 2022 [71]	Case Report	Australia (Oceania)	Neonate with congenital HPeV meningitis was born in poor condition and with petechiae. MRI on day 2 of life demonstrated diffuse subcortical white matter abnormality
Clinical Presentation Laboratory Imaging	Lim SMJ, 2022 [72]	Case Report	Japan (Asia)	At birth the neonate with congenital HPeV3 meningoencephalitis was irritable and at 9 h of age, febrile with repeated episodes of desaturation, bradycardia, and abnormal limb movements. CSF showed no pleocytosis, normal levels of glucose and slightly elevated levels of proteins. Brain MRI performed on day 2 of life demonstrated white matter inflammatory changes; EEG was compatible with seizure activity
Clinical Presentation Laboratory Imaging Therapy Outcome	Salavati S, 2020 [73]	Case Report	The Netherlands (Europe)	A preterm neonate with congenital HPeV meningoencephalitis, after birth became bradycardic, pale, hypotonic. He was intubated and ventilated for 5 days. Pleocytosis was found in CSF. MRI demonstrated bilateral ischemic injury, restricted diffusion in white matter, corpus callosum, corticospinal tract, pulvinar and optic radiation. EEG showed subclinical seizures. Antibiotic and antiepileptic therapy was administered. At 2 years follow up the patient presented cerebral visual impairment, cognitive, language and internalising behaviour problems
Laboratory Outcome	Izumita R, 2019 [74]	Original Research	Japan (Asia)	Pleocytosis was absent in all 16 CSF samples. 1 out of 16 patients had a severe neuro-psychomotor retardation at a 3 year follow up
Laboratory	Sugiura K, 2020 [75]	Case Report	Japan (Asia)	Higher levels of IL-6, IL-17 and TNF alpha were found in the serum of patients affected by HPeV meningitis compared with controls
Laboratory	Habuka R, 2020 [76]	Original Research	Japan (Asia)	Higher serum level of proinflammatory cytokine/chemokine was present in HPeV infected patients than in Enterovirus infected patient
Imaging	Sarma A, 2019 [77]	Retrospective case series	USA (America)	Head ultrasound had normal findings in 2/6 patients. All cases had brain MRI exhibiting diffuse and multifocal subcortical white matter involvement
Imaging	Lane LM, 2021 [78]	Case Report	Ireland (Europe)	An infant boy with HPeV meningoencephalitis had normal head ultrasound. Brain MRI showed extensive diffusion abnormalities in the deep white matter, corpus callosum, deep grey nuclei and midbrain. EEG showed multiregional seizure onset
Therapy	Rhoden E, 2020 [79]	Original research	USA (America)	Posaconazole inhibits HPeV-A3 infection by directly targeting the capsid and interfering with virus-cell interactions.
Outcome	De Ceano-Vivas M, 2021 [80]	Original research	Spain (Europe)	At a 18 months follow up, 3 out of 15 (20%) patients presented developmental concern
Outcome	De Crom SC, 2021 [81]	Multicenter prospective cohort study	the Netherlands (Europe)	At 24 months follow-up, 3 children with previous HPeV infection, including a case of meningitis, had impaired motor development
Outcome	Martin del Valle F, 2019 [82]	Original Research	Spain (Europe)	At 2 years follow up 1 out of 15 patients presented with a gross motor dysfunction
Outcome	Britton PN, 2018 [83]	Cohort study	Australia (Oceania)	At 12 months following hospitalised HPeV infection, 5% of infants showed severe neurological sequelae, 23% moderate, 11% mild and 61% had a normal neurodevelopment. Neurological impairment mainly interested the gross motor domain
Outcome	Britton PN, 2020 [84]	Cohort study	Australia (Oceania)	At 3 years following hospitalised HPeV infection, all children showed age-appropriate development on BSID-III. The lowest scores were in the gross motor domain, but still in the normality range

## Data Availability

Data sharing is not applicable to this article as no datasets were generated or analysed during the current study.

## Abbreviations

HPeV: Human parechovirus
PRISMA: Preferred Reporting Items for Systematic review and Meta Analysis
CNS: Central Nervous System
CSF: Cerebrospinal Fluid
LDH: Lactate dehydrogenase
EV: Enterovirus
IL: interleukin
TNF: tumour necrosis factor
WBCc: white blood cell count
WBC: white blood cell
ALT: alanine aminotransferase
AST: aspartate aminotransferase
RBC: red blood cell
CRP: C reactive protein
CT: computerised tomography
MRI: Magnetic resonance imaging
EEG: Electroencephalography
IQR: interquartile range
BSID-III: Bayley Scales of Infant and Toddler Development, Third Edition
GMF: gross motor function

## References

[1] Ancora G, Faldella G, Chiereghin A, Marsico C, Nigro CS, Lazzarotto T, Sambri V, Brusa G, Capretti MG (2020). Parechovirus Infection causing sepsis-like Illness in newborns: a NICU approach. New Microbiol.

[2] Tricco AC, Lillie E, Zarin W, O’Brien KK, Colquhoun H, Levac D, Moher D, Peters MDJ, Horsley T, Weeks L, Hempel S, Akl EA, Chang C, McGowan J, Stewart L, Hartling L, Aldcroft A, Wilson MG, Garritty C, Lewin S, Godfrey CM, Macdonald MT, Langlois EV, Soares-Weiser K, Moriarty J, Clifford T, Tunçalp Ö, Straus SE (2018). PRISMA Extension for scoping reviews (PRISMA-ScR): Checklist and Explanation. Ann Intern Med.

[3] Ouzzani M, Hammady H, Fedorowicz Z, Elmagarmid A (2016). Rayyan-a web and mobile app for systematic reviews. Syst Rev.

[4] Page MJ, McKenzie JE, Bossuyt PM, Boutron I, Hoffmann TC, Mulrow CD, Shamseer L, Tetzlaff JM, Akl EA, Brennan SE, Chou R, Glanville J, Grimshaw JM, Hróbjartsson A, Lalu MM, Li T, Loder EW, Mayo-Wilson E, McDonald S, McGuinness LA, Stewart LA, Thomas J, Tricco AC, Welch VA, Whiting P, Moher D (2021). The PRISMA 2020 statement: an updated guideline for reporting systematic reviews. BMJ.

[5] Naccache SN, Lustestica M, Fahit M, Mestas J, Dien Bard J (2018). One year in the life of a Rapid Syndromic Panel for Meningitis/Encephalitis: a Pediatric Tertiary Care Facility’s experience. J Clin Microbiol.

[6] Abedi GR, Messacar K, Luong W, Nix WA, Rogers S, Queen K, Tong S, Oberste MS, Watt J, Rothrock G, Dominguez S, Gerber SI, Watson JT (2019). Picornavirus etiology of acute Infections among hospitalized infants. J Clin Virol.

[7] Lee BR, Sasidharan A, Harrison CJ, Selvarangan R (2020). Positive impact of routine testing for enterovirus and parechovirus on length of hospitalization and antimicrobial use among inpatients ≤ 6 months of age. J Clin Microbiol.

[8] Tomatis Souverbielle C, Wang H, Feister J, Campbell J, Medoro A, Mejias A, Ramilo O, Pietropaolo D, Salamon D, Leber A, Erdem G (2021). Year-Round, routine testing of multiple body site specimens for human parechovirus in Young Febrile infants. J Pediatr.

[9] Sasidharan A, Banerjee D, Harrison CJ, Selvarangan R (2021). Emergence of Parechovirus A3 as the Leading cause of Central Nervous System Infection, surpassing any single enterovirus type, in children in Kansas City, Missouri, USA, from 2007 to 2016. J Clin Microbiol.

[10] Singh SH, Hassouneh L. Parechovirus in infancy: a 5-year experience at a pediatric center. The American Journal of the Medical Sciences; 2023.

[11] Black S, Bradley C, Lai FY, Shenoy S, Bandi S, Allen DJ, Tang JW (2019). Comparing the clinical severity of Disease caused by enteroviruses and human parechoviruses in neonates and infants. Pediatr Infect Dis J.

[12] Ferreras Antolín L, Kadambari S, Braccio S, Tang JW, Xerry J, Allen DJ, Ladhani SN (2018). Parechovirus Surveillance Network. Increased detection of human parechovirus Infection in infants in England during 2016: epidemiology and clinical characteristics. Arch Dis Child.

[13] Cosgun Y, Bayrakdar F, Karademirtok H, Atak T, Korukluoglu G. Role of viral agents in Childhood Central Nervous System Infections. Journal of Pediatric Infectious Diseases; 2020.

[14] Chakrabarti P, Warren C, Vincent L, Kumar Y (2018). Outcome of routine cerebrospinal fluid screening for enterovirus and human parechovirus Infection among infants with sepsis-like Illness or Meningitis in Cornwall, UK. Eur J Pediatr.

[15] Sirin MC, Goktas S (2018). Determination of the prevalence of viral, bacterial and fungal pathogens causing Meningitis by using multiplex real-time polymerase chain reaction. Acta Med Mediterranea.

[16] Bal A, Saz EU, Arslan SY, Atik S, Bayturan S, Yurtseven A, Gazi H, Cicek C, Kurugol Z, Bal ZS. The evaluation of the diagnostic performance of the BioFire FilmArray Meningitis/Encephalitis Panel in children: a retrospective Multicenter Study. J Pediatr Infect Dis. 2022.

[17] de Jong EP, van den Beuken MGA, van Elzakker EPM, Wolthers KC, Sprij AJ, Lopriore E, Walther FJ, Brus F (2018). Epidemiology of Sepsis-like Illness in Young infants: major role of Enterovirus and Human Parechovirus. Pediatr Infect Dis J.

[18] Eichinger A, Hagen A, Meyer-Bühn M, Huebner J (2019). Clinical benefits of introducing real-time multiplex PCR for cerebrospinal fluid as routine diagnostic at a tertiary care pediatric center. Infection.

[19] Elling R, Böttcher S, du Bois F, Müller A, Prifert C, Weissbrich B, Hofmann J, Korn K, Eis-Hübinger AM, Hufnagel M, Panning M (2019). Epidemiology of human parechovirus type 3 upsurge in 2 hospitals, Freiburg, Germany, 2018. Emerg Infect Dis.

[20] Posnakoglou L, Siahanidou T, Syriopoulou V, Michos A (2020). Impact of cerebrospinal fluid syndromic testing in the management of children with suspected central nervous system Infection. Eur J Clin Microbiol Infect Dis.

[21] Posnakoglou L, Tatsi EB, Siahanidou T, Syriopoulou V, Michos A (2021). Genetic variations in human parechovirus type 3 in infants with central nervous system Infection. Virol Sin.

[22] Vincent JJ, Zandotti C, Baron S, Kandil C, Levy PY, Drancourt M, Raoult D, Ninove L (2020). Point-of-care multiplexed diagnosis of Meningitis using the FilmArray® ME panel technology. Eur J Clin Microbiol Infect Dis.

[23] Marchand S, Launay E, Schuffenecker I, Gras-Le Guen C, Imbert-Marcille BM, Coste-Burel M (2021). Severity of parechovirus Infections in infants under 3 months of age and comparison with enterovirus Infections: a French retrospective study. Arch Pediatr.

[24] Schnuriger A, Vimont S, Godmer A, Gozlan J, Gallah S, Macé M, Lalande V, Saloum K, Perrier M, Veziris N, Morand-Joubert L. Differential Performance of the FilmArray Meningitis/Encephalitis Assay To Detect Bacterial and Viral Pathogens in Both Pediatric and Adult Populations. Microbiology Spectrum. 2022. 10.1128/spectrum.02774-21 PMC9045182 35404096

[25] Bucci S, Coltella L, Martini L, Santisi A, De Rose DU, Piccioni L, Campi F, Ronchetti MP, Longo D, Lucignani G, Dotta A, Auriti C (2022). Clinical and neurodevelopmental characteristics of *Enterovirus* and *Parechovirus* Meningitis in neonates. Front Pediatr.

[26] Stephens C, Reynolds C, Cremin M, Barry R, Morley U, Gibson L, De Gascun CF, Felsenstein S (2021). Parent-administered neurodevelopmental follow up in children after Picornavirus CNS Infections. Pediatr Infect Dis J.

[27] Sano K, Hamada H, Hirose S, Sugiura K, Harada S, Koizumi M, Hara M, Nishijima H, Taira M, Ogura A, Ogawa T, Takanashi JI (2018). Prevalence and characteristics of human parechovirus and enterovirus Infection in febrile infants. Pediatr Int.

[28] Abe Y, Ohno T, Matsumoto H, Daimon Y, Kurahashi H, Takayama R, Sakaguchi Y, Tanabe S, Tanaka F, Miyamoto Y, Kawano A, Yamanouchi H (2021). HPeV3-associated acute encephalitis/encephalopathy among Japanese infants. Brain Dev.

[29] Izumita R, Deuchi K, Aizawa Y, Habuka R, Watanabe K, Otsuka T, Saitoh A (2019). Intrafamilial transmission of Parechovirus A and enteroviruses in neonates and Young infants. J Pediatr Infect Dis Snamfooc.

[30] Suzuki Y, Aizawa Y, Izumita R, Habuka R, Watanabe K, Saitoh A (2021). PCR detection rates for serum and cerebrospinal fluid from neonates and young infants infected with human parechovirus 3 and enteroviruses. J Clin Virol.

[31] Park SE, Song D, Shin K, Nam SO, Ko A, Kong J, Kim YM, Yeon GM, Lee YJ (2019). Prospective research of human parechovirus and cytokines in cerebrospinal fluid of young children less than one year with sepsis-like Illness: comparison with enterovirus. J Clin Virol.

[32] Park SE, Lim TJ, Nam SO, Chang CL, Byun SY, Ko A, Kong J, Cho JW, Yeon GM, Lee YJ (2021). Clinical utility of the FilmArray meningitis/encephalitis panel in children at a tertiary center in South Korea. Brain Dev.

[33] Rhie S. Clinical differences between enterovirus and human parechovirus in children and infants. Annals of Child Neurology. 2020.

[34] Nam EJ, Ham JY, Song KE, Kim YK, Lee NY. Incidence and distribution of the pathogens causing Central Nervous System Infections at the University Hospital of Korea. Clin Lab. 2021;67(6). 10.7754/Clin.Lab.2020.201118 34107628

[35] Chang JT, Chen YS, Chen BC, Huang TS, Chang TH (2018). Human parechovirus Infection in children in Taiwan: a Retrospective, single-hospital study. Jpn J Infect Dis.

[36] Nassrallah B, Bamberger E, Cohen S, Srugo I, Golan-Shany O, Shlonsky Y, Mubariki R, Genizi J (2021). The yield of CSF molecular testing in febrile neonates. Eur J Clin Microbiol Infect Dis.

[37] Mathew S, Al Khatib HA, Al Ansari K, Nader J, Nasrallah GK, Younes NN, Coyle PV, Al Thani AA, Al Maslamani MA, Yassine HM (2021). Epidemiology Profile of viral Meningitis Infections among patients in Qatar (2015–2018). Front Med (Lausanne).

[38] O’Brien MP, Francis JR, Marr IM, Baird RW (2018). Impact of Cerebrospinal Fluid Multiplex assay on diagnosis and outcomes of Central Nervous System Infections in children: a before and after Cohort Study. Pediatr Infect Dis J.

[39] Berkhout A, Cheng DR, McNab S, Lee LY, Daley AJ, Clifford V (2023). Clinical and Health System Impact of Biofire Filmarray Meningitis/Encephalitis Routine Testing of CSF in a Pediatric Hospital: an observational study. Pediatr Infect Dis J.

[40] Chamings A, Druce J, Caly L, Yoga Y, Britton PN, Macartney KK, Alexandersen S (2019). Evolutionary analysis of human parechovirus type 3 and clinical outcomes of Infection during the 2017-18 Australian epidemic. Sci Rep.

[41] Chamings A, Liew KC, Reid E, Athan E, Raditsis A, Vuillermin P, Yoga Y, Caly L, Druce J, Alexandersen S (2019). An Emerging Human Parechovirus Type 5 Causing Sepsis-Like Illness in infants in Australia. Viruses.

[42] Abdelrahim NA, Mohammed N, Evander M, Ahlm C, Fadl-Elmula IM (2022). Viral Meningitis in Sudanese children: differentiation, etiology and review of literature. Med (Baltim).

[43] Fourgeaud J, Mirand A, Demortier J, Kamus L, Collet L, Olivier S, Henquell C, Vauloup-Fellous C (2022). Enterovirus Meningitis in Mayotte French Comoros Island, March-June 2019. J Clin Virol.

[44] Garrido R, Antunes J, Pedroso H, Fialho M, Cunha M. Parechovirus neonatal sepsis and Meningitis – a (still) poorly recognised agent. J Pediatr Neonatal Individualized Med. 2022.

[45] Fox B, Sabio Paz V, Incardona MA, Elisiri ME, Gonzalez Fraga S, Solana CL, Fernández-Canigia L. Rapid syndromic molecular testing and human parechovirus Infection in children: a report of three cases in Argentina. Rev Argent Microbiol. 2022 Jan-Mar;54(1):31–4. 10.1016/j.ram.2021.02.003 33838970

[46] Alhazmi A, Lazrek M, Alidjinou EK, Engelmann I, Schuffenecker I, Dubos F, Hober D (2020). Repeated viral Meningitis in a newborn. J Neurovirol.

[47] Chowdhury SR, Lee D (2020). Atypical acral swelling and viral exanthem with parechovirus Meningitis. J Paediatr Child Health.

[48] Berk MC, Bruning AHL, van Wassenaer-Leemhuis AG, Wolthers KC, Pajkrt D (2018). Human parechovirus Meningitis with adverse neurodevelopmental outcome: a Case Report. Pediatr Infect Dis J.

[49] Piralla A, Perniciaro S, Ossola S, Giardina F, De Carli A, Bossi A, Agosti M, Baldanti F (2019). Human parechovirus type 5 neurological Infection in a neonate with a favourable outcome: a case report. Int J Infect Dis.

[50] Yehia R, Weidow N, Maertens PA, Tengsupakul S. Successful treatment of parechovirus meningoencephalitis and Myocarditis in two neonates. The American Journal of the Medical Sciences; 2023.

[51] Kirkley MJ, Robinson C, Dominguez SR, Messacar K (2019). Neonatal parechovirus Infection mimicking a surgical abdomen. BMJ Case Rep.

[52] Kanagaratnam M, Jyothish D. Parechovirus Meningitis in infants-time for routine CSF viral screening? Archives of Disease in Childhood; 2019.

[53] Urooj F, Flinders P, Paul J, Padmakumar B. A case series of human parechovirus associated sepsis and Meningitis in young children. Arch Dis Child. 2019.

[54] Lee D, Loh SW, Tan J, Chong J (2018). Acral-accentuated exanthem in an infant with parechovirus Meningitis. Pediatr Dermatol.

[55] Masanori T, Endo A, Hisata K, Kudo T, Shimizu T (2022). Parechovirus Infection in an infant with severe abdominal distention. Pediatr Int.

[56] Tokmak DN, Yalçınkaya R, Gülenç N, Öz FN, Kaman A, Teke TA, Durmuş SY, Tanır G (2021). Parechovirus Infection mimicking bacterial sepsis in an infant. J Pediatr Inf.

[57] Tokak S, Özdemir M, Gülseren YD, Çaksen H (2021). A case of human parechovirus Infection in an infant with Meningitis. J Pediatr Inf.

[58] Tierradentro-García LO, Zandifar A, Kim JDU, Andronikou S (2022). Neuroimaging findings in Parechovirus Encephalitis: a Case Series of Pediatric patients. Pediatr Neurol.

[59] van Hinsbergh T, Elbers RG, Bouman Z, van Furth M, Obihara C (2022). Neurodevelopmental outcomes of newborns and infants with parechovirus and enterovirus central nervous Infection: a 5-year longitudinal study. Eur J Pediatr.

[60] van Hinsbergh TMT, de Crom SCM, Lindeboom R, van Furth MAM, Obihara CC (2019). Human parechovirus Meningitis and gross-motor neurodevelopment in young children. Eur J Pediatr.

[61] Tan JHY, Choong CT, Tee NWS, Chong CY, Thoon KC, Maiwald M, Lee EYX, Tan MSS, Tan NWH (2022). Clinical profile of children with parechovirus Meningitis in Singapore. J Neurovirol.

[62] Linhares MI, Brett A, Correia L, Pereira H, Correia C, Oleastro M, De Sousa R, Rodrigues F (2021). Parechovirus Genotype 3 outbreak among Young infants in Portugal. Acta Med Port.

[63] Kielar M, Tokarz A, Dumnicka P, Maraj M, Burzyńska B, Stępniewski S (2019). Parechovirus and enterovirus Infections in neonates. Folia Med Cracov.

[64] Samarasekara H, Janto C, Balgahom R, Polkinghorne A, Branley J (2020). Unexpected detection of human parechovirus in infants with suspected Meningitis using real-time multiplex PCR. Pathology.

[65] Silcock RA, Doyle R, Clark JE, Kynaston JA, Thomas M, May ML (2022). Parechovirus Infection in infants: evidence-based parental counselling for paediatricians. J Paediatr Child Health.

[66] Joseph L, May M, Thomas M, Smerdon C, Tozer S, Bialasiewicz S, McKenna R, Sargent P, Kynaston A, Heney C, Clark JE (2019). Human parechovirus 3 in infants: expanding our knowledge of adverse outcomes. Pediatr Infect Dis J.

[67] McKenna R, Joseph L, Sargent P, May M, Tozer S, Bialasiewicz S, Heney C, Schlapbach LJ, Clark JE (2019). Paediatric intensive care admissions during the 2015–2016 Queensland human parechovirus outbreak. J Paediatr Child Health.

[68] Midgley CM, Jackson MA, Selvarangan R, Franklin P, Holzschuh EL, Lloyd J, Scaletta J, Straily A, Tubach S, Willingham A, Nix WA, Oberste MS, Harrison CJ, Hunt C, Turabelidze G, Gerber SI, Watson JT (2018). Severe parechovirus 3 Infections in Young infants-Kansas and Missouri, 2014. J Pediatr Infect Dis Soc.

[69] Kadambari S, Braccio S, Ribeiro S, Allen DJ, Pebody R, Brown D, Cunney R, Sharland M, Ladhani S (2019). Enterovirus and parechovirus Meningitis in infants younger than 90 days old in the UK and Republic of Ireland: a British Paediatric Surveillance Unit study. Arch Dis Child.

[70] Roh DE, Kwon JE, Kim YH (2020). Human parechovirus: an emerging cause of sepsis-like syndrome in infants aged under 3 months. Pediatr Infect Vaccine.

[71] Hilbig A, Liew KC, Foster C, Fuller DG, Chamings A, Alexandersen S (2022). Neonatal parechovirus Infection: possibility of in-utero transmission. J Paediatr Child Health.

[72] Mei Jy Lim S, Foley DA, Lakshmanan R, Caly L, Gehlot S, Davis J (2022). A case of congenital human parechovirus type 3 Meningoencephalitis. Pediatr Infect Dis J.

[73] Salavati S, Salavati M, Coenen MA, Ter Horst HJ, Bos AF (2020). A parechovirus type 3 Infection with a presumed Intrauterine Onset: a poor neurodevelopmental outcome. Neonatology.

[74] Izumita R, Aizawa Y, Watanabe K, Saitoh A (2019). Persistence of high neutralizing antibody titers after neonatal and early infantile Infection with Parechovirus-A3. Pediatr Infect Dis J.

[75] Sugiura K, Ogura A, Takanashi JI, Hamada H (2020). Multiple hypercytokinemia in human parechovirus-3 Infection in infants with pediatric systemic inflammatory response syndrome. Chiba Med J.

[76] Habuka R, Aizawa Y, Izumita R, Domon H, Terao Y, Takihara H, Okuda S, Saitoh A (2020). Innate Immune responses in serum and Cerebrospinal Fluid from neonates and infants infected with Parechovirus-A3 or enteroviruses. J Infect Dis.

[77] Sarma A, Hanzlik E, Krishnasarma R, Pagano L, Pruthi S (2019). Human parechovirus meningoencephalitis: neuroimaging in the era of polymerase chain reaction-based testing. AJNR Am J Neuroradiol.

[78] Lane LM, McDermott MB, O’Connor P, Cronly S, O’Regan M, De Gascun CF, Morley U, Snow A, Tone S, Heffernan J, Cryan JB. Multicystic Encephalomalacia: the neuropathology of systemic neonatal parechovirus Infection. Pediatr Dev Pathol. 2021 Sep-Oct;24(5):460–6. 10.1177/10935266211001645 33754905

[79] Rhoden E, Ng TFF, Campagnoli R, Nix WA, Konopka-Anstadt J, Selvarangan R, Briesach L, Oberste MS, Weldon WC (2020). Antifungal triazole posaconazole targets an early stage of the parechovirus A3 life cycle. Antimicrob Agents Chemother.

[80] de Ceano-Vivas M, García ML, Velázquez A, Del Martín F, Menasalvas A, Cilla A, Epalza C, Romero MP, Cabrerizo M, Calvo C (2021). Neurodevelopmental outcomes of infants younger than 90 days old following enterovirus and parechovirus Infections of the Central Nervous System. Front Pediatr.

[81] de Crom SC, van Hinsbergh MT, van Beijsterveldt IA, van Furth AM, Rossen JW, Obihara CC (2023). Motor development of children after a human parechovirus or enterovirus Infection: 24 months follow-up. Minerva Pediatr (Torino).

[82] Del Martín F, Menasalvas Ruiz A, Cilla A, González AV, de Ceano Vivas M, Cabrerizo Sanz M, Calvo C (2019). Neurodevelopment medium-term outcome after parechovirus Infection. Early Hum Dev.

[83] Britton PN, Khandaker G, Khatami A, Teutsch S, Francis S, McMullan BJ, Jones CA (2018). High prevalence of developmental concern amongst infants at 12 months following hospitalised parechovirus Infection. J Paediatr Child Health.

[84] Britton PN, Walker K, McMullan B, Galea C, Burrell R, Morgan B, Honan I, Teutsch S, Smithers-Sheedy H, Fairbairn N, Mattick R, Hutchinson D, Jones CA (2020). Early life parechovirus Infection neurodevelopmental outcomes at 3 years: a Cohort Study. J Pediatr.

